# Gestational weight gain charts for twin pregnancies in Southeast China

**DOI:** 10.1186/s12884-020-2761-1

**Published:** 2020-02-24

**Authors:** Lihua Lin, Juan Lin, Xiaodan Mao, Libo Xu, Ronghua Zhang, Jinying Luo, Yingying Lin, Jianying Yan

**Affiliations:** 1 Department of Healthcare, Fujian Provincial Maternity and Children’s Hospital, Affiliated Hospital of Fujian Medical University, Fuzhou, 350001 Fujian Province People’s Republic of China; 2 Department of Obstetrics, Fujian Provincial Maternity and Children’s Hospital, Affiliated Hospital of Fujian Medical University, 18 Daoshan Road, Fuzhou, 350001 Fujian Province People’s Republic of China; 3 Laboratory of Gynecologic Oncology, Fujian Provincial Maternity and Children’s Hospital, Affiliated Hospital of Fujian Medical University, Fuzhou, 350001 Fujian People’s Republic of China; 4 Department of Computer Technology, Fujian Provincial Maternity and Children’s Hospital, Affiliated Hospital of Fujian Medical University, Fuzhou, 350001 Fujian Province People’s Republic of China

**Keywords:** Gestational weight gain, Twin pregnancies, Hierarchical linear regression models, Body mass index

## Abstract

**Background:**

To establish age-standardized charts of weight gain for term twin pregnancies in Southeast China.

**Methods:**

We designed a retrospective study on data from women pregnant with twins, a gestational age beyond 36 weeks and an average weight ≥ 2500 g. We established hierarchical linear regression models to express gestational weight gain patterns.

**Results:**

We analyzed data from 884 women pregnant with twins (151 underweight, 597 normal weight, and 136 overweight). Our final models fit the crude weight measurement data well. The means of weight gain generally decreased as the pre-pregnancy BMI increased. For each BMI category, the mean weight gains increased with the gestational age and the standard deviation increased slightly. The mean weight gains were 18.82 ± 6.73, 18.53 ± 6.74, and 16.97 ± 6.95 kg at 37 weeks in underweight, normal weight, and overweight women, respectively.

**Conclusion:**

The weight gain chart can be used to estimate maternal weight gain to be gestational age–standardized z scores by pre-pregnancy BMI and may serve as an innovative tool for perinatal care providers to guide the weight gain of women pregnant with twins.

## Background

Gestational weight gain is an important indicator of the nutritional status of pregnant women and is strongly associated with the maternal and fetal growth health [1, 2]. Regardless of the pre-pregnancy body mass index (BMI), inappropriate gestational weight gains including insufficient and excessive gestational weight gains are closely associated with short- and long- term maternal and child health outcomes [3]. Excessive gestational weight gain increases the risks of preeclampsia, gestational diabetes, postpartum weight retention, large gestation age newborns, childhood obesity, and even excessive gestational weight gain in a subsequent pregnancy; while insufficient gestational weight gain may result in preterm births, fetal growth restriction, or stillbirths [1, 4–7].

Twin pregnancies are associated with higher risks of adverse pregnancy outcomes than singleton pregnancies [8–10]. In 1990, the Institute of Medicine (IOM) introduced guidelines for gestational weight gain in twin pregnancies so as to lower the risks of adverse pregnancy outcomes. These were later updated in 2009, on the basis of the recorded interquartile range of weight gain from a study of females delivering twins after more than 36 weeks of gestation [1]. However, these guidelines for twin pregnancies have been questioned and have been referred to as “provisional standards” [11, 12]. Moreover, studies analyzing these guidelines have presented conflicting results demonstrating that women who gain weight beyond the IOM guidelines achieve healthy pregnancy outcomes [12, 13]. Studies have also indicated that excessive weight gain in may improve neonatal birth weight without worsening maternal outcomes [11, 13, 14].

Gestational weight gain and adverse events in twin pregnancies are often confounded with the length of gestation. Majority adverse events like pre-term birth or still birth occur with reduced gestational age on account of which mothers do not gain adequate weight. It is therefore difficult to analyze if preterm birth leads to low gestational weight or reduced weight of the mother contributes to premature birth. Despite high morbidity and mortality in twin pregnancies, few studies have examined the optimal gestational weight gain for such females and there have been no clear guidelines [12–16].

A gestational weight gain z-score chart has been proposed as a tool to establish an unbiased direct association between pregnancy weight gain and perinatal outcomes [17, 18]. The chart describes the gestational age-standardized mean and the standard deviations (SDs) throughout gestation, providing a precise tool to monitor the pattern of weight gain and to initiate appropriate health interventions [18]. This chart has received global attention, including in Sweden and the United States [17, 19, 20]. However, to the best of our knowledge, only one study, conducted in the US, has established age-standardized weight gain charts for dichorionic twins [21]. Their recommendations may not be suitable for women in the Chinese population due to differences in ethnicity, culture, and dietary habits. Therefore, the objective of this study was to establish age-standardized charts of weight gain for women throughout their twin pregnancies in Southeast China.

## Methods

### Study population

We retrospectively reviewed medical records of women who received perinatal care and delivered twins at the Fujian Provincial Maternity and Children’s Hospital between January 2013 and November 2019. We analyzed data from women if they met the following inclusion criteria: (1) both twins were alive at birth; (2) the delivery gestational age was not lower than 37 weeks; (3) the average twin birth weight was ≥2500 g; (4) the mothers had no chronic diseases, such as diabetes or hypertension diagnosed before pregnancy; and (5) they had complete medical records with pre-pregnancy weight, heigth, twin birth weights, gestational age, and weight measurements within 1 week of delivery. We excluded women with twin pregnancies that had been reduced from multiple pregnancies or those whose weight records were missed.

As this was a retrospective study that did not involve interactions with the study women or data that could be used to identify them, the Ethics Committee of the Fujian Provincial Maternity and Children’s Hospital approved it and waived the requirement for informed consents.

### Study variables

We extracted demographic information and maternal characteristics, including age, education, pre-pregnancy weight, height, pre-pregnancy BMI, parity, and gestational age at delivery from the hospital’s electronic medical records. The medical records also contained detailed information from each perinatal examination, medical diagnoses, and neonatal data. The women were weighed wearing light clothes at each perinatal visit using an electronic weighing scale; the weight measurements were accurate to 0.1 kg. We calculated pre-pregnancy BMIs (in kg/m^2^) based on the self-reported pre-pregnancy weights and heights. We defined delivery weight as the weight at delivery or within 1 week of delivery. The gestational weight gain equaled the maternal weight at delivery minus the pre-pregnancy weight. We calculated the maternal weight gain at various weeks by the weekly-measured weight minus the pre-pregnancy weight. We used the Chinese standard to classify the pre-pregnancy BMIs [pre-pregnancy weight (kg)]/[height-squared (m^2^)] into following categories: underweight (< 18.5 kg/m^2^), normal weight (18.5–23.99 kg/m^2^), overweight (24.0–27.9 kg/m^2^), and obese (≥28.0 kg/m^2^) [22]. The gestational ages had been estimated from the last menstrual period and were confirmed by ultrasonography during the first trimester.

### Statistical analysis

We reported continuous variables as means ± SDs and categorical variables as numbers (percentages) to describe the baseline characteristics. We performed one-way analysis of variances (ANOVA) for continuous variables and Chi-squared analysis for categorical variables to compare the baseline characteristics of the women between pre-pregnancy BMI groups. We also performed multiple comparison (Scheffe method) to assess the differences between every two groups if the result of ANOVA or Chi-squared analyses were statistically significant. We considered *P* < 0.05 as statistically significant. We applied hierarchical linear regression models to describe the gestational age-related maternal weight gain during pregnancy in each pre-pregnancy BMI group. We first determined the normality of the distribution of repeated weight gain measurement data. We built normal Q-Q plots showing good-fit normal distributions (see Additional file 1: Figure S1). Next, we performed restricted cubic splines to model gestational age to obtain smooth and non-linear weight gain curves [23]. The knots of the restricted cubic splines based on the use of Akaike information criterion and combined the available evidence of the model of Hutcheon et al. [21].We regarded individuals as random effects and added them to the intercept and linear term of restricted cubic splines to correlate between and within women’s repeated prenatal weight measurements. Based on the final regression equations, we derived and created charts of weight gain means, percentiles, and SDs of maternal weight gain at each gestational age according to the pre-pregnancy BMI categories. Then, we superimposed scatter diagrams on the estimated lines to assess the goodness-of-fit model by checking the proportion of crude data falling within the threshold of mean ± 1SD and mean ± 2SD. Finally, we compared the IOM guidelines and the recommended range in the established z-score charts. The recommended range was the weight gain corresponding to the interquartile range (P25-P75) of the z-score that was calculated in study subjects. We conducted all modeling using the R software (version 3.4.2), and performed all other statistical analyses using the SPSS software (version 12.0; SPSS, Chicago, IL, USA).

## Results

### Baseline characteristics

**Fig. 1:**
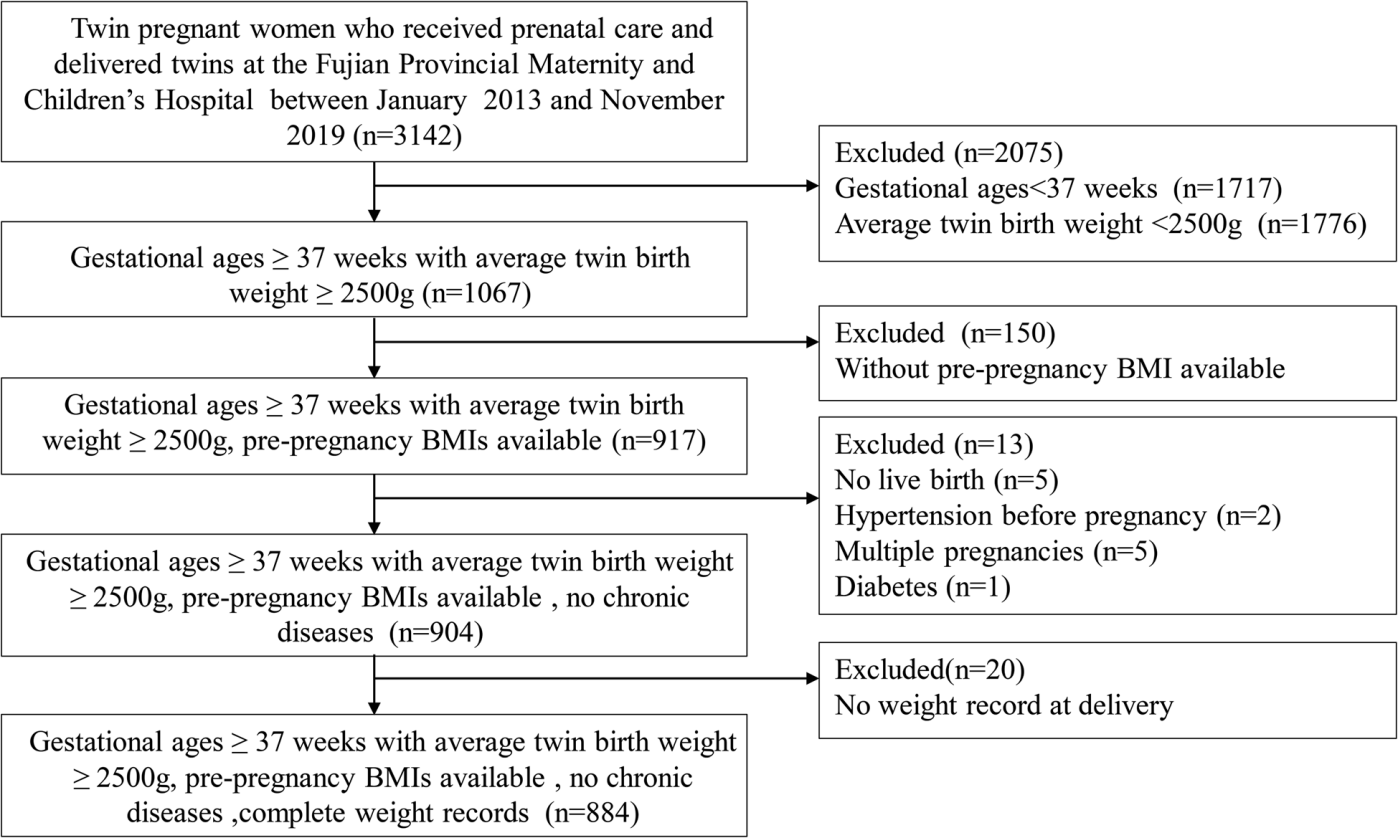
Flowchart for 884 women with twin pregnancies who delivered their babies at the Fujian Provincial Maternity and Children’s Hospital between January 2013 and November 2019

A total of 3142 pregnant women received perinatal care and delivered twins at the Fujian Provincial Maternity and Children’s Hospital between January 2013 and November 2019. We excluded data from the following women: 2075 twin pregnant women who delivered before 37 weeks or whose average twin birth weights were lower than 2500 g, 150 women with missing pre-pregnancy BMIs, 5 women with stillbirths, 5 women having been reduced from multiples, 1 woman with diabetes, 2 woman with hypertension, and 20 women without delivery weight records. We used data from the remaining 884 pregnant women (Fig. 1). Based on their pre-pregnancy BMIs, 151 (17.1%) women were underweight, 597 (63.1%) were normal weight, and 120 (13.6%) were overweight, 16 (1.80%) women were obese.As the small number of obse women, we merged them into overweight group. Table 1 shows the baseline characteristics. The average maternal ages increased across pre-pregnancy BMI categories, 28.2 ± 4.1 years in underweight women, 30.3 ± 4.2 years in normal weight women, and 30.7 ± 4.3 years in overweight women (*P* < 0.001). The average height was 160.9 ± 5.0 cm. The average gestational age at delivery was 37.8 ± 0.7 weeks. Approximately one-fifth of women delivered monochorionic twins. Nearly half of the women in each group were nulliparous without significant differences in the proportions among groups. The average birth weight was 2815.8 ± 253.2 g and increased as the pre-pregnancy BMIs increased. The total weight gains decreased as the pre-pregnancy BMIs increased (*P* < 0.001), i.e., the underweight women had significantly greater total weight gains (19.5 ± 4.6 kg) than the normal weight women (19.0 ± 5.1 kg) and overweight women (17.4 ± 5.6 kg). We found no significant differences in maternal heights, educational years among the three groups. According to the 2009 IOM guidelines, in the normal weight group, 34.0% of women had low gestational weight gains, 51.4% had normal gestational weight gains, and 14.6% had high gestational weight gains. In the overweight women group, 31.6% of women had low gestational weight gains, 50.7% had normal gestational weight gains, and 17.6% had high gestational weight gains. We could not categorize these values in underweight women because a recommendation for gestational weight gains for this group is lacking from the 2009 IOM guidelines.

**Table 1:** Baseline characteristics of women pregnancies who delivered their babies at the Fujian Provincial Maternity and Children’s Hospital between January 2013 and November 2019

Characteristics	All participants	Underweight	Normal weight	Overweight	*P* value
Total numbers	884	151	597	136	
Maternal age (years)	30.0 ± 4.3	28.2 ± 4.1	30.3 ± 4.2	30.7 ± 4.3	< 0.001^a^
Maternal height (cm)	160.9 ± 5.0	160.9 ± 4.4	161.1 ± 5.1	160.3 ± 5.2	0.309
Education (years)	12.8 ± 3.3	12.5 ± 3.1	13.0 ± 3.3	12.2 ± 3.3	0.033 ^a^
Gestational age at delivery (weeks)	37.8 ± 0.7	37.9 ± 0.8	37.8 ± 0.7	37.7 ± 0.6	0.058
Monochorionic twins	164(18.6)	36(23.8)	109(18.3)	19(14.0)	0.094
Nulliparity	514(58.1)	87 (57.6)	352 (59.0)	75 (55.1)	0.711
Average birth weight (g)	2815.8 ± 253.2	2793.0 ± 247.6	2810.2 ± 255.5	2865.2 ± 244.6	0.035 ^a^
Total weight gain (kg)	18.8 ± 5.1	19.5 ± 4.6	19.0 ± 5.1	17.4 ± 5.6	0.001 ^a^
GWG below guidelines	–	–	203 (34.0)	43 (31.6)	0.641
GWG within guidelines	–	–	307 (51.4)	69 (50.7)	
GWG above guidelines	–	–	87 (14.6)	24 (17.6)	

^a^ *P* value is statistically significant between any two groups

### Fitting gestational weight gain models

**Fig. 2:**
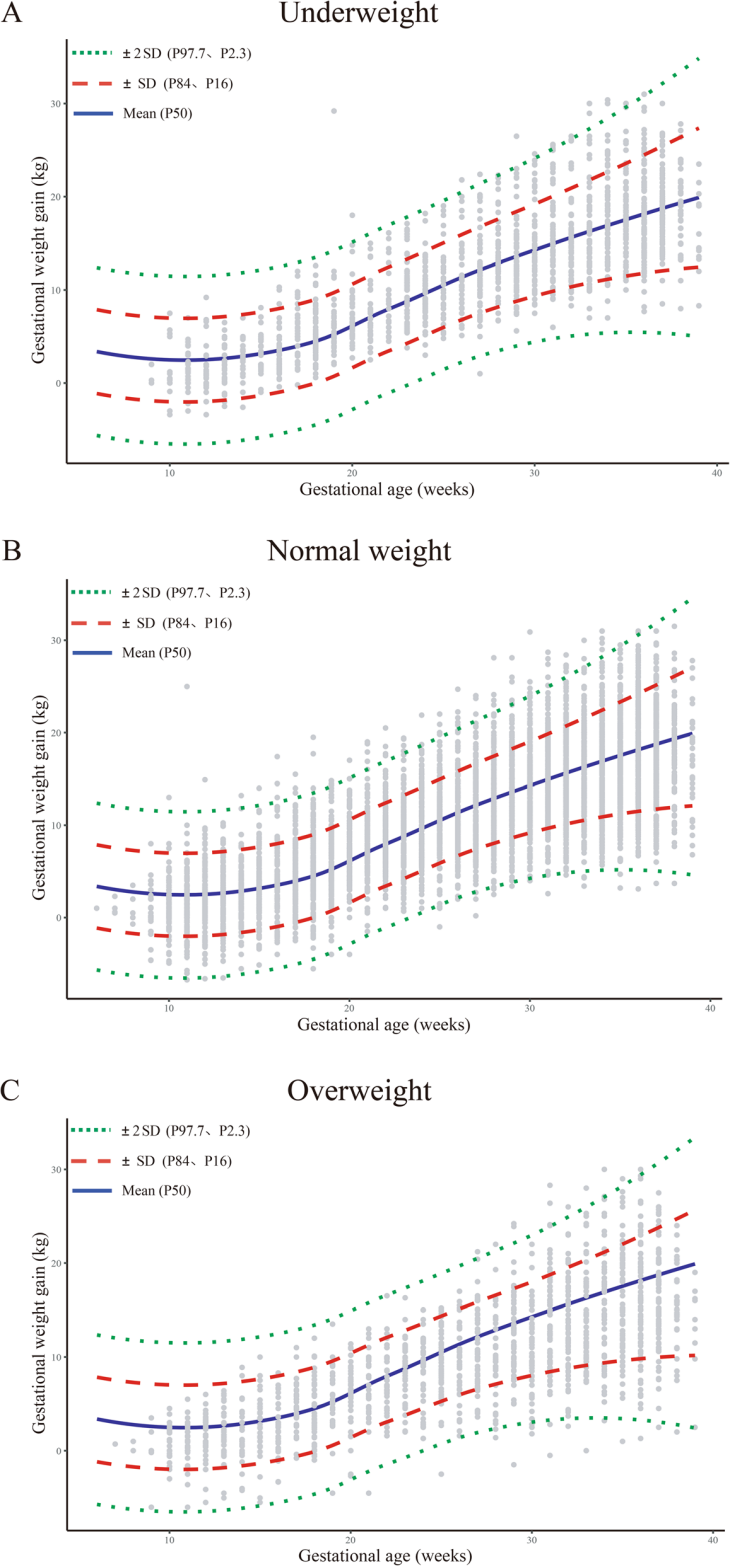
Estimated lines from hierarchical linear regression superimposed on crude weight measurements for women with twin pregnancies at the Fujian Provincial Maternity and Children’s Hospital who delivered between January 2013 and November 2019 Grey dots indicate the crude weight measurements. Lines denote the fitted mean, mean ± 1SD, and mean ± 2SD.

Table 2 and Fig. 2 shows the equations (for the estimated means and SDs) and final model lines (for mean, mean ± 1 SD and mean ± 2 SD lines) superimposed on the crude weight measurements in each BMI category. We chose the number and the location of the spline knots based on the model fit to gain the lower Akaike Information Criterion (AIC).We compared the fit of model with 4 knots and 5 knots in restricted cubic spline and our final models used five selected knots at 9, 20, 28, 33, and 37 weeks of gestation for restricted cubic splines. We isolated the resulting variance components from the model to present the correlations between and within women using fractional polynomials (data not shown). Our final models fit the crude weight measurements data well, with 71.6, 78.6, and 69.2% of crude observations falling within the mean ± 1 SD threshold (68% expected), and 93.9, 98.2, and 95.5% falling within the mean ± 2 SD threshold (95% expected) in the underweight, normal weight, and overweight BMI categories, respectively.

**Table 2:** Multilevel linear regression equations for means and SDs of pregnancy weight gain for gestational ages in underweight, normal-weight, and overweight groups

BMI category	Regression equation
Underweight	
Mean	2.867073 + 3.597614 × GA Spline term_1_–12.54554 × GA Spline term_2_+ 19.79477 × GA Spline term_3_–22.73170 × GA Spline term_4_
SD	Sqrt[16.108 + 0.04079 (GA Spline term_1_^2) + 4.132]
Normal weight	
Mean	2.867073 + 3.568180 × GA Spline term_1_–12.49818 × GA Spline term_2_+ 19.7948319.79483 × GA Spline term_3_–22.73137-22.73137 × GA Spline term_4_
SD	Sqrt[16.10812+ 0.0410957 (GA Spline term_1_^2) + 4.132]
Overweight	
Mean	2.867073 + 3.357253 × GA Spline term_1_–12.09662 × GA Spline term_2_+ 19.79483 × GA Spline term_3_–22.73169× GA Spline term_4_
SD	Sqrt[16.17164+ 0.0457317 (GA Spline term_1_^2) + 4.132]

*GA* gestational age; *sqrt* square root

The gestational weight gains followed a nonlinear trajectory in each BMI category with a flat pattern of weight gain in underweight and normal weight women, but weight loss in overweight women, in the first trimester. Table 3 lists the values of smoothed age-standardized means and SDs of weight gain. As expected, the means of weight gain on women with same gestational ages, generally decreased as the pre-pregnancy BMI increased. For example, the mean weight gains were 12.67 ± 4.72 kg for underweight women, 12.53 ± 4.72 kg for normal weight women, and 11.61 ± 4.75 kg for overweight women at 28 weeks; and 18.82 ± 6.73 kg for underweight women, 18.53 ± 6.74 kg for normal weight women, and 16.97 ± 6.95 kg for overweight women at 37 weeks. In addition, the age-specific gestational weight gain means increased with the gestational age and the SD increased slightly. Using the mean and the SD, we calculated the z-score: z = (actual weight gain − mean weight gain)/SD. For example, the z-score of a normal weight pregnant woman with twins who gains 15 kg at 28 weeks was calculated as (15.00–12.53)/4.72 = 0.52, where the 12.53 and 4.72were the mean and SD of weight gain. A z-score of zero corresponds to the mean of weight gain, and a z-score below or above zero would indicate a weight gain below or above the average. Deviations of the z-score from zero reflect a deviation away from the mean [24].

**Table 3:** Means and standard deviations (SDs) of gestational weight gain for gestational ages in underweight, normal-weight, and overweight groups based on regression equations

Gestational Age	Underweight			Normal weight			Overweight		
	Weight observations	Mean (kg)	SD	Weight observations	Mean (kg)	SD	Weight observations	Mean (kg)	SD
7	0	NA	NA	4	0.61	4.53	1	−0.79	4.54
8	0	NA	NA	6	0.94	4.53	1	−0.40	4.54
9	3	1.40	4.53	23	1.27	4.53	4	0.50	4.54
10	13	1.73	4.53	94	1.61	4.53	23	1.02	4.54
11	26	2.07	4.53	155	1.94	4.53	33	1.61	4.54
12	28	2.42	4.53	148	2.29	4.53	43	2.26	4.54
13	26	2.87	4.53	133	2.66	4.53	32	2.87	4.54
14	29	2.91	4.53	147	2.91	4.53	28	2.91	4.54
15	27	3.02	4.53	147	3.02	4.53	34	3.01	4.54
16	40	3.24	4.53	136	3.23	4.53	35	3.21	4.54
17	30	3.59	4.53	192	3.58	4.53	42	3.54	4.54
18	55	4.11	4.53	236	4.10	4.53	48	4.03	4.54
19	34	4.84	4.53	165	4.83	4.53	31	4.71	4.54
20	42	5.79	4.54	131	5.77	4.54	37	5.60	4.54
21	31	6.90	4.54	141	6.87	4.54	30	6.63	4.55
22	29	8.08	4.54	187	8.04	4.54	42	7.72	4.55
23	52	9.24	4.55	202	9.19	4.55	42	8.78	4.56
24	48	10.30	4.57	194	10.23	4.57	38	9.73	4.58
25	34	11.18	4.59	177	11.09	4.59	47	10.49	4.60
26	51	11.81	4.62	210	11.71	4.62	51	11.00	4.64
27	56	12.27	4.66	222	12.15	4.66	55	11.34	4.68
28	48	12.67	4.72	244	12.53	4.72	49	11.61	4.75
29	69	13.10	4.80	285	12.95	4.80	65	11.93	4.84
30	57	13.68	4.91	267	13.51	4.91	57	12.39	4.96
31	74	14.47	5.05	309	14.28	5.05	75	13.07	5.11
32	74	15.39	5.22	313	15.18	5.22	67	13.89	5.30
33	69	16.33	5.43	296	16.10	5.44	72	14.74	5.54
34	89	17.17	5.69	374	16.93	5.70	78	15.50	5.82
35	87	17.84	6.00	379	17.58	6.01	91	16.10	6.16
36	120	18.37	6.35	465	18.09	6.36	106	16.57	6.54
37	102	18.82	6.73	409	18.53	6.74	96	16.97	6.95
38	41	19.27	7.13	109	18.96	7.15	35	17.37	7.39
39	12	19.71	7.55	27	19.39	7.57	6	17.76	7.85

Weight gains for any desired percentile can be calculated: the percentile for gestational weight gain = mean + k SD, where k is the z-score corresponding to a particular percentile; for example, k = 1.64 for the 95th and − 1.64 for 5th percentiles. However, because of the limited sample size, we mainly focused on the model and gestational weight gain z-score chart in normal weight women, but only presented estimated means and SDs values in underweight and overweight women. We generated the chart for normal weight women containing the gestational age-standardized weight gains in selected percentiles (that is, 3rd, 5th, 10th, 50th, 90th, 95th, and 97th) using mean and SD equations (Table 4).

**Table 4:** Age-standardized means, standard deviations (SDs), and selected percentiles of gestational weight gain in normal weight women who delivered at the Fujian Provincial Maternity and Children’s Hospital between January 2013 and November 2019

Gestational Age	3^rd^	5^th^	10^th^	50^th^	90^th^	95^th^	97^th^
7	-7.91	-6.82	-5.19	0.61	6.41	8.04	9.13
8	-7.58	-6.49	-4.86	0.94	6.74	8.37	9.46
9	-7.25	-6.16	-4.53	1.27	7.07	8.70	9.79
10	-6.91	-5.82	-4.19	1.61	7.41	9.04	10.13
11	-6.58	-5.49	-3.86	1.94	7.74	9.37	10.46
12	-6.23	-5.14	-3.51	2.29	8.09	9.72	10.81
13	-5.86	-4.77	-3.14	2.66	8.46	10.09	11.18
14	-5.61	-4.52	-2.89	2.91	8.71	10.34	11.43
15	-5.50	-4.41	-2.78	3.02	8.82	10.45	11.54
16	-5.29	-4.20	-2.57	3.23	9.03	10.66	11.75
17	-4.94	-3.85	-2.22	3.58	9.38	11.01	12.10
18	-4.42	-3.33	-1.70	4.10	9.90	11.53	12.62
19	-3.69	-2.60	-0.97	4.83	10.63	12.26	13.35
20	-2.77	-1.68	-0.04	5.77	11.58	13.22	14.31
21	-1.67	-0.58	1.06	6.87	12.68	14.32	15.41
22	-0.50	0.59	2.23	8.04	13.85	15.49	16.58
23	0.64	1.73	3.37	9.19	15.01	16.65	17.74
24	1.64	2.74	4.38	10.23	16.08	17.72	18.82
25	2.46	3.56	5.21	11.09	16.97	18.62	19.72
26	3.02	4.13	5.80	11.71	17.62	19.29	20.40
27	3.39	4.51	6.19	12.15	18.11	19.79	20.91
28	3.66	4.79	6.49	12.53	18.57	20.27	21.40
29	3.93	5.08	6.81	12.95	19.09	20.82	21.97
30	4.28	5.46	7.23	13.51	19.79	21.56	22.74
31	4.79	6.00	7.82	14.28	20.74	22.56	23.77
32	5.37	6.62	8.50	15.18	21.86	23.74	24.99
33	5.87	7.18	9.14	16.10	23.06	25.02	26.33
34	6.21	7.58	9.63	16.93	24.23	26.28	27.65
35	6.28	7.72	9.89	17.58	25.27	27.44	28.88
36	6.13	7.66	9.95	18.09	26.23	28.52	30.05
37	5.86	7.48	9.90	18.53	27.16	29.58	31.20
38	5.52	7.23	9.81	18.96	28.11	30.69	32.40
39	5.16	6.98	9.70	19.39	29.08	31.80	33.62

### Comparison with previous studies and IOM recommendations

We evaluated the range of total weight gain at term in twin pregnant women with normal weight study subjects using the percentile method. As shown in Table 5, the interquartile range (P25–P75) of total gestational weight gain in our data from women with normal weight was 15.3–22.5 kg. The corresponding gestational weight gains (interquartile range) of our z-score chart and another published z-score chart [21] in the women with normal weight were 15.1–23.0 and 19.6–25.5 kg, respectively. Compared with the IOM recommendation for normal weight women (16.8–24.5 kg), the values of the lower and upper limit of the corresponding ranges for interquartile z-score values of our z-score chart and interquartile range (P25–P75) of total gestational weight gain were both lower than those of the IOM recommendation range. Compared with the published z-scores of Hutcheon et al. [21], the lower and upper limits of weight gain in our z-score chart were lower, but closer to those in the IOM recommendation range. It is worth noting that the corresponding gestational weight gain in our z-score chart fell within the IOM recommendation range.

**Table 5:** Comparison of values of weight gain in normal-weight women established by the percentile method for different measures

GWG measure	25th	75th	Interquartile range	Corresponding ranges (kg)
Total GWG	15.3	22.5	15.3~22.5	15.3~22.5
Z-score A^a^	0.61	1.63	0.61~1.63	15.1~23.0
Z-score B^b^	−0.83	0.34	−0.83~0.34	19.6~25.5
IOM recommendation	16.8-	24.5	16.8–24.5	16.8–24.5

*GWG* gestational weight gain

^a^ z-score chart of normal-weight women established in our study

^b^ z-score chart of normal-weight women established by Hutcheon et al.

## Discussion

We created gestational weight gain z-score charts for twin pregnant women in Southeast China for three pre-pregnancy BMI categories. The gestational weight gain has a significant influence on maternal and infant health outcomes and has attracted the attention of obstetricians and researchers. The 2009 revised IOM guidelines on twin gestational weight gains have been questioned for having been based on a single historic study using descriptive data [16]. Numerous researchers have sought to examine the 2009 IOM guidelines for twin pregnancies, and few studies have corroborated the conclusion that weight gains meeting or exceeding the lower limit in the guidelines are more significantly associated with improved outcomes than the insufficient weight gains [25], and no consensus has been reached. Total gestational weight gains are commonly associated with pregnancy outcomes. However, pregnancy outcomes are not only associated with total gestational weight gain but depend on the duration of gestation; that is, women with shorter gestational ages have fewer chances to gain weight than women with longer gestational ages and may suffer adverse pregnancy outcomes such as preterm birth, which may lead false correlations between gestational weight gain and pregnancy outcomes [26]. Z-score charts can overcome these shortcomings and can estimate gestational weight gain independently of gestational ages. They provide statistical percentiles and parameters (means and SDs) to calculate z-scores. The charts can also help researchers compare differences in weight gain between pregnant women at different gestational ages. The charts of gestational weight gain z-scores at term for women with singleton pregnancy have become well established, but the data for twins remain limited. Moreover, such z-score charts have not yet been established in Chinese populations since the implementation of the new birth policy and the wide use of assisted reproductive technology, both of which have contributed to increasing the rates of twin pregnancies. To fill this gap, we established weight gain charts for twin pregnancies containing reference values for gestational weight gains. The charts show weight gain patterns and developed standards using statistical methods to control for the gestational age, as well as accounting for repeated measurements between women in situations when adjustment for gestational age in a multivariable model may be inappropriate. The weight gains across pregnancies observed in our study showed similar trajectories to those published for twins weight gain z-score charts restricted to uncomplicated dichorionic twin pregnancies [21].

We analyzed only data from women with twin pregnancies and gestational ages of at least 37 weeks and average live-birth weights not smaller than 2500 g as the “healthy group” based on the 2009 IOM twin guidelines. The final model fit the observed data well and the gestational weight gains in our z-score charts increased over the course of pregnancy. In contrast with previous z-score studies, where weight measurement data showed a non-normal distribution and log-transformed response variables and models [17, 19–21, 27], our weight measurement data followed a normal distribution. However, the weight gain over gestational ages in all of these studies, including ours, followed curvilinear trajectories. We observed similar weight gain patterns in the twin pregnant population in another study [21] and in the three pre-pregnancy BMI groups in our study population. However, the weight gain means in the normal weight women at 37, 38, and 39 weeks were 18.5,18.9, and 19.4 kg, respectively, in our study; these values were lower than those obtained by Hutcheon et al. [21]. Similarly, for overweight pregnant women, our average cumulative weight gain was 17.4 kg at 38 weeks, and it was lower than their result of 19.1 kg [21]. Moreover, Barbara et al. found that the total weight gains in twins were in the range of 22.7–28.1 kg for underweight women, 18.1–24.5 kg for normal weight women, and 17.2–21.3 kg for overweight women [28].These discrepancies may be explained by the different populations selected. The published standard chart for twins was based on analysis of a US population with relatively loose inclusion and exclusion criteria for dichorionic twin pregnant women beyond 35 weeks’ gestation [21]. These standard charts may not describe optimal weight gain patterns because recommended delivery gestational age for twins is between 37 and 42 weeks with average twin birth weights ≥2500 g to reduce mortality and morbidity. In contrast, we created our charts for developing appropriate weight gains as a guideline for term twin pregnancies in China. Moreover, the interquartile ranges of the total gestational weight gains in normal weight women in our study were lower than those of the IOM guidelines and those of other studies conducted in Northern China [29]. This discrepancy may be attributed to differences in culture, economics, customs, and perinatal care advice. Women in the US are substantially taller than Asian women [30]. In our research population, the average height of women was nearly 161 cm, shorter than the 165 cm in the research cohort of Barbara et al. [28] and than the 163 cm in the research cohort by Hutcheon et al. [21], suggesting that their weight gain models and z-score charts are not generalizable to the Chinese population.

When compared with the IOM recommendations, the corresponding weight gain range fell close to the recommended IOM range, suggesting that our charts may serve as references in practice. However, the patterns of gestational weight gain in our study accounted only for term pregnancies and twin average weight outcomes. Gestational weight gain is associated with other short- and long-term adverse pregnancy outcomes, including postpartum weight retention and childhood obesity. Therefore, future studies should be carried out with more representative samples to confirm associations of gestational weight gain z-scores with maternal and infant health outcomes. With well implemented health programs, gestational weight gain z-score charts may be used as an important tool to monitor weight gain and reduce adverse pregnancy outcomes associated with inappropriate weight gains. Finally, exploration of other statistical techniques for a more sophisticated gestational weight gain model would be worthwhile.

Our study involved women seeking perinatal care at the Fujian Provincial Maternity and Children’s Hospital, and this was the first study generating z-scores for twin gestations in Southeast China; these z-score charts are meaningful and should be useful to evaluate maternal weight gains during twin gestations, especially in the Fujian provinces.

We are aware of some limitations of our study. First, the study data were not population-based, but were drawn from data on women with twin pregnancies receiving perinatal care at one hospital, possibly leading to poor representation of the general population. These women may have had a better education or higher economic status than those in the general population, contributing to higher healthcare awareness. Another limitation of the study is the small sample size; we did not have a sufficient number of cases to establish sound z-score charts for underweight, overweight, and obese women. Further studies are needed to develop the z-score charts of other BMI groups across multiple centers. Finally, as mentioned in most studies on gestational weight gain [19–21, 27], we used self-reported weights rather than measured weights prior to pregnancy, because pre-pregnancy weights are often not available in medical records or research studies [31, 32]; this may have resulted in miscalculations of gestational weight gain or misclassification of pre-pregnancy BMIs. For this reason, the lack of weight measurements during the first trimester may have affected the construction and accuracy of our models.

## Conclusions

This is the first production of gestational weight gain z-score charts for twin pregnancies in China. Our weight gain charts according to gestational ages express the total weight gain to age-standardized z-scores, which are independent of gestational ages and can be used for clinical and epidemiological purposes to identify risks of adverse pregnancy outcomes. Gestational weight gains increased across pregnancy in each pre-pregnancy BMI group and were lower in women with higher pre-pregnancy BMIs than in those with lower pre-pregnancy BMIs. Future studies with larger populations should be conducted to produce sound and reliable z-score gestational weight gain recommendations.

## Supplementary information

**Additional file 1.** Q-Q plots of normality distributions of weight gain observations for women with twin pregnancies at the Fujian Provincial Maternity and Children’s Hospital who delivered between January 2013 and November 2019

## Data Availability

The datasets used and/or analyzed during the current study are available from the corresponding author on reasonable request.

## Abbreviations

ANOVA: Analysis of variances
BMI: Body mass index
IOM: Institute of medicine
SD: Standard deviation
