# Supplementary figures and images for: Gestational weight gain charts for twin pregnancies in Southeast China
Source: BMC Pregnancy Childbirth. 2020 Feb 24;20:127. doi: 10.1186/s12884-020-2761-1 (PMC7041099; doi:10.1186/s12884-020-2761-1)

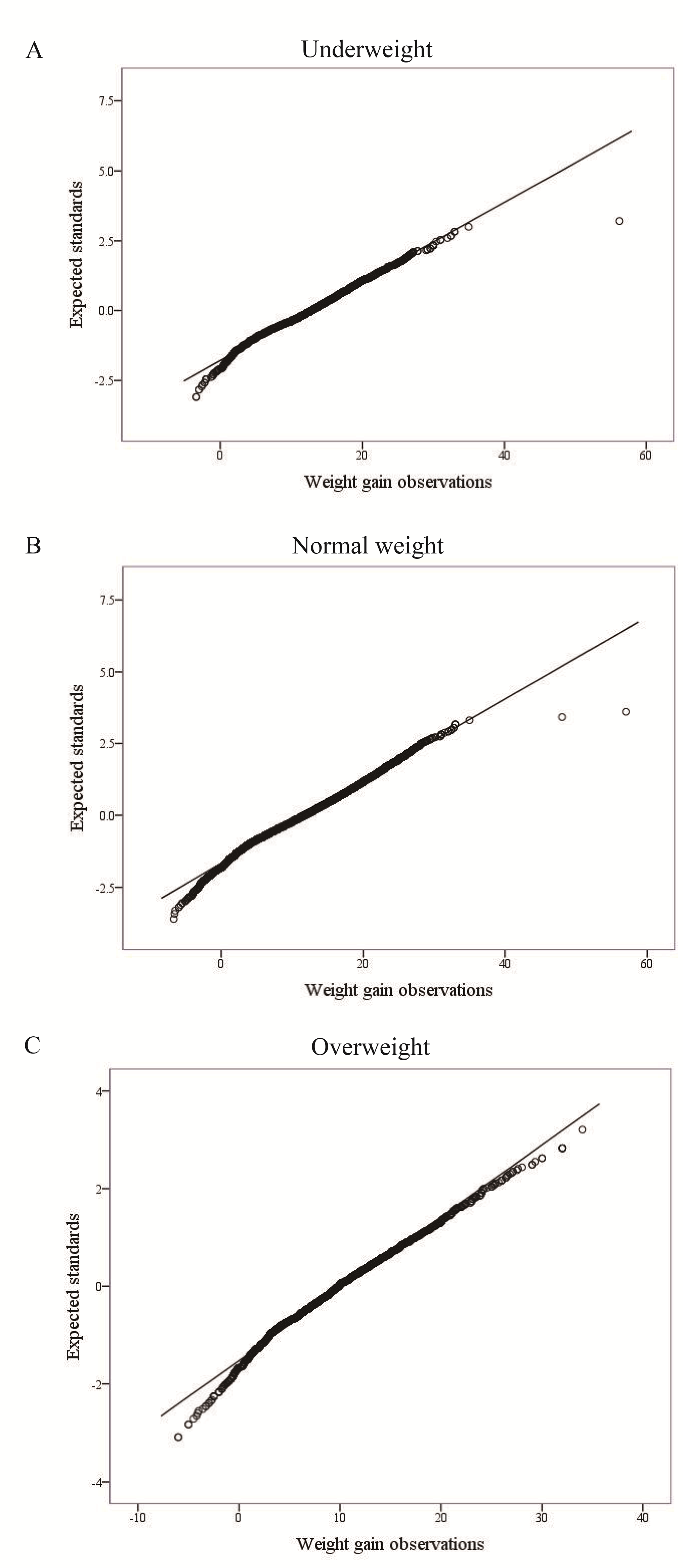

Supplement: Supplementary file 1 — Additional file 1. Q-Q plots of normality distributions of weight gain observations for women with twin pregnancies at the Fujian Provincial Maternity and Children’s Hospital who delivered between January 2013 and November 2019 [file 12884_2020_2761_MOESM1_ESM.tif]
